# Priority setting in the German healthcare system: results from a discrete choice experiment

**DOI:** 10.1007/s10754-023-09347-y

**Published:** 2023-05-15

**Authors:** V. Meusel, E. Mentzakis, P. Baji, G. Fiorentini, F. Paolucci

**Affiliations:** 1 Faculty of Medicine, FAU Erlangen-Nürnberg, Erlangen, Germany; 2 Department of Economics, City University of London, Northampton Square, London, EC1V 0HB UK; 3 Department of Health Economics, Corvinus University of Budapest, Budapest, Hungary; 4 Department of Economics, University of Bologna, Bologna, Italy; 5 Sir Walter Murdoch School of Public Policy and International Affairs, Murdoch University, Perth, WA Australia; 6 Department of Sociology and Law & Economics, University of Bologna, Bologna, Italy

**Keywords:** Decision making, Priority setting, Efficiency, Discrete choice experiment, Healthcare, Innovation, Germany, I1

## Abstract

Worldwide, social healthcare systems must face the challenges of a growing scarcity of resources and of its inevitable distributional effects. Explicit criteria are needed to define the boundaries of public reimbursement decisions. As Germany stands at the beginning of such a discussion, more formalised priority setting procedures seem in order. Recent research identified multi-criteria decision analysis (MCDA) as a promising approach to inform and to guide decision-making in healthcare systems. In that regard, this paper aims to analyse the relative weight assigned to various criteria in setting priority interventions in Germany. A discrete choice experiment (DCE) was employed in 2015 to elicit equity and efficiency preferences of 263 decision makers, through six attributes. The experiment allowed us to rate different policy interventions based on their features in a composite league table (CLT). As number of potential beneficiaries, severity of disease, individual health benefits and cost-effectiveness are the most relevant criteria for German decision makers within the sample population, the results display an overall higher preference towards efficiency criteria. Specific high priority interventions are mental disorders and cardiovascular diseases.

## Introduction

Priority setting in healthcare decision-making is inevitable. Limited health resources, growing health expenditures, combined with technological advances and an ageing population, continue to put pressure on healthcare systems (Fleck, 2001; Emanuel, 2000; Fuchs, 2010, Borck et al., 2012). Policymakers are encouraged to consider priority setting in healthcare, albeit such rationing decisions may not always be based on transparent processes, but are ad-hoc (Baltussen & Niessen, 2006) or history-based (Kapiriri & Martin, 2007), possibly leading to a suboptimal use of resources.

Complex developments in healthcare are widening the gap between new technological possibilities and advances in therapy and diagnosis and the financial feasibility of the German Healthcare system (Borck, 2012; di Costanzo, 2020). In that regard, concepts such as rationalisation and prioritisation are intensely debated in the quest to implement an equitable allocation of resources (Schmitz-Luhn & Katzenmeier, 2016).

For Germany, the controversial debate on healthcare expenditures and limits on the availability of healthcare services offered by the system started relatively late compared to other countries (Sabik & Lie, 2008).^1^ Germany’s health expenditures have steadily increased from 9.4 per cent of its Gross Domestic Product (GDP) in 1992–11.7% in 2019, placing Germany in 6th place in the world rank and on top of the EU27 (8,3%) (OECD, 2021, Statistische Bundesamt, 2022). In international terms however, the German healthcare system stands out for a generous benefit package with high levels of capacity and relatively low cost-sharing (Beske & Drabinski, 2005). As in most high income countries, the future levels of expenditure on GDP, due to demographic and epidemiologic changes (e.g. increase in life expectancy, a diminishing mortality rate) (Institute for Health Metrics & Evaluation, 2016; World Bank, 2016) combined with the increasing costs of medicine are likely to be significantly higher. This has implications for the statutory health insurance benefits as the Federal Joint Committee (G-BA) decides over what adequate, appropriate, economic healthcare entails (Federal Joint Committee, 2017). However, rationalisation in terms of efficiency gains alone will hardly be enough to prevent a further divergence between the feasible and the financially viable.

The complexity of such decisions suggests the need for explicit criteria to be used (Alliance for Health Policy & Systems Research, 2004; Baltussen & Niessen, 2006; Chalkidou et al., 2016; Cromwell et al., 2015). In this respect, discrete choice experiments (DCEs) allow for the simultaneous assessment of multiple policy criteria and preferences elicitation of decision-makers when facing trade-off decisions (de Bekker-Grob et al., 2012; Hansen & Devlin, 2019; Lancsar & Louviere, 2008; Ryan & Gerard, 2003; Thokala et al., 2016).

Past studies support the feasibility and acceptability of DCEs in eliciting public preferences towards societal values and attribute-driven interventions (Genie et al., 2020; Green & Gerard, 2009; Krauth et al., 2021; Krinke et al., 2019; López-Bastida et al., 2019; Luyten et al., 2015). Empirical studies in Germany eliciting priority rankings for the treatments of determined groups, find treatments for children are ranked highest (Raspe & Stumpf, 2013) whereas Diederich (2011) finds little evidence that the German public accepts age as a priority criterion for healthcare services, although there is wide agreement to prioritise specific age groups.

A combination of efficiency and societal values tends to be predominantly considered in priority setting (Baeten et al., 2010; Kenny & Joffres, 2008). With the advent of numerous new initiatives in the health sector, decision-makers are expected to choose between competing healthcare interventions and explicitly consider equity and efficiency trade-off (Baltussen & Niessen, 2006). Transparent and informed decision-making in healthcare requires national level criteria are to be established (Mitton & Donaldson, 2004; Ottersen et al., 2016; WHO, 2007) encouraging a more open social and political discourse on the priorities in medical care to guide policy making on which healthcare technologies should be publicly financed at the different levels of the Social Health Insuracne (SHI) system (Diederich, ; Gerst, 2014; Norheim, 2016).

This paper presents the results of a DCE conducted in 2015, aiming to explore key stakeholders’ preferences for different features of healthcare policies and interventions in Germany, and to show how the latter are prioritised according to such preferences. The paper builds on past work (Defchereux et al., 2012; Mirelman et al., 2012; Mentzakis et al., 2014; Paolucci et al., 2015; Baji et al., 2016) and extends the pool of countries with available data for cross country comparisons.

## Background on the German health care system

The main pillar of the German system is the Statutory Health Insurance which is inspired by strong solidarity principles providing the normative basis to pursue the objectives of equity and comprehensiveness, and which represents the framework of regulations on the provision and the financing of healthcare services (Oduncu, 2013). The SHI regulations aim for cost-containment and sustainable financing mechanisms, managed competition, as well as improved efficacy and quality (Blümel & Busse, 2017; Sauerland, 2001).

At the federal level, the Social Code Book V gives a foundation for entitlements, rights and duties of insureds covered by SHI. However, it does not lay down specific guidelines but, instead, sets a framework for policy interfaces (Dannecker, 2009). The scope of benefits is subject to negotiations between the latter bodies and the associations of payers and providers (Blümel et al., 2020). In an international comparison, the German benefit catalogue shows to be quite extensive, leading to reform efforts and the question about how far the solidarity should go. The Reform Act in 2004 showed early attempts of rationing certain benefits from the catalogue, for instance medications for the treatment of erectile dysfunction, hair loss or smoking cessation (Burkhardt, 2012).

Ever since its resumption, the various aspects of prioritisation have been discussed following different political strategies and institutional procedures (Friedrich et al., 2009; Groß et al., 2010; Heil et al., 2010; Müller & Groß, 2009; Oduncu, 2012; Peacock et al., 2006; Raspe & Meyer, 2009; Schöne-Seifert, 2006, Heyers, 2016, Petri, 2015). The Central Ethics Committee for Observance of Ethical Principles in Medicine (ZEKO) and the German Medical Association have promoted the concept of prioritisation (ZEKO, 2007; ZEKO, 2000; Bundesärztekammer, 2014; Borck et al., 2012; Raspe & Schulze, 2013; Diederich et al., 2011) with specific focus on its legal, ethical and economic aspects and have supported the use of pre-defined criteria to evaluate medical services and benefits (Marckmann, 2009; Gordijn & Have, 2013; Oduncu, 2012; Borck et al., 2012; Storz & Egger, 2010; Kliemt, 2006).

As such, the three major criteria of prioritisation *‘‘medical need’*’ (severity and urgency), *‘‘proven benefit and fitness for purpose’*’, and *‘‘cost–benefit-effectiveness’*’ have been proposed by the central ethics committee (ZEKO, 2007).

## Methodology

A discrete choice experiment (DCE) was employed to assess the relative weight of various criteria in setting priorities in the German health arena. DCE are commonly used in healthcare for prioritisation decisions (Lancsar & Louviere, 2008; Ryan & Gerard, 2003; Ryan et al., 2008) using public preferences. Health economists have acknowledged the benefit of the approach especially when facing health policy, planning and resource allocation decisions in high-income countries. In that regard DCEs are widely applied to a range of policy questions (Baji et al., 2016; de Bekker-Grob et al., 2012; Ryan & Gerard, 2003; Whitty et al., 2011) and priority setting frameworks (Baltussen et al., 2006; Peacock et al., 2010; Razavi et al., 2020). These include the elicitation of views on diagnosis, treatment and care (van de Schoot et al., 2017, Koopmanschap et al., 2010, Clark et al., 2017, King et al., 2007; Kjaer & Gyrd-Hansen, 2008), access to services (Longo et al., 2006; Mengoni et al., 2013), consumer (health) preferences (Czoli et al., 2016) and the employment preferences of health personnel (Mandeville et al., 2014; Wordsworth et al., 2004).

Respondents’ preferences are elicited in a survey adapted from previous studies (Defechereux et al., 2012; Mirelman et al., 2012; Paolucci et al., 2015). Respondents’ were asked to choose among a set of hypothetical alternative interventions presented in choice sets, with each alternative described in terms of six criteria. To every criterion, values have been assigned over a range of pre-defined levels.

### Experimental and instrument design

In the first stage, the decision-making context was defined and a set of key attributes was narrowed down accordingly. Here, a combination of relevant efficiency/equity-related factors was included, where efficiency is mainly referred to as the maximisation of health gains within society at lowest cost, including non-health outcomes. Equity criteria, on the other hand, are related to the distributional effects of interventions, aiming for the reduction of inequalities in health status or targeting disadvantaged groups (James et al., 2005).

An existing standardized questionnaire reported earlier for other countries (Baltussen et al., 2006; Koopmanschap et al., 2010; Mirelman et al., 2012) was used comprising a core set of preference criteria as attribute. Those have been identified based on literature reviews and were verified by national focus groups of health programmers and experts within the initial three settings. These were in Ghana, Nepal, as well as a working session with 28 leading HTA experts at the HTAi conference in 2008 (Baeten et al., 2010; Baltussen et al., 2007; Defechereux et al., 2012; Mirelman et al., 2012; Noorani et al., 2007; Paolucci et al., 2015; Tanios et al., 2013).

Overall, six attributes have been identified as comprising key criteria in health decision making for our DCE: one with three levels and five with two levels (Table 1). This set of criteria describes the most generic aspects of a health intervention. The chosen criteria were grouped under the equity (willingness to subsidise others, severity of disease, age of the target group) and efficiency (number of beneficiaries, cost-effectiveness, individual health benefits) categories. Further, the selected attributes have been consistent with those used in previous studies in which they proved to be important preference criteria for priority setting (Baltussen et al., 2010).

**Table 1:** Definition of Attributes and Levels

Criteria (Attribute)	Levels	Definition
Severity of Disease	Not severe^a^	The intervention would treat a condition with life expectancy of 2 or more life years without the intervention
	Severe	Intervention would treat a condition with life expectancy of less than 2 life years without the intervention
Number of Potential Beneficiaries	Few^a^	Less than 100,000 persons will benefit from intervention
	Many	More than 100,000 persons will benefit from intervention
Age of Target Group	Young^a^	Targeting young individuals between 0 and 14 years
	Middle-Age	Targeting middle-aged individuals between 15 and 59 years
	Elderly	Targeting elderly individuals over 60 years
Individual Health Benefits	Small^a^	Less than 5 healthy life years on average per individual within the target group
	Large	More than 5 healthy life years gained on average per individual within the target group
Willingness to subsidize others	Less than 70%^a^	Of healthcare services expenditures will be covered through collective pooling (e.g. subsidies)
	More than 70%	Of healthcare services expenditures will be covered through collective pooling (e.g. subsidies)
Cost-effectiveness	Cost-effective	The intervention is cost effective—cost per DALY is less than the GDP per capita [Cost-effectiveness threshold values according to WHO, cost/DALY < 1*GDP/capita = very cost- effective, cost/DALY = 1–3*GDP/capita = cost-effective, cost/DALY > 3*GDP/capita = not cost- effective (WHO 2005)]
	Not cost-effective^a^	The intervention is not cost effective (cost per DALY is more than the GDP per capita

^a^Reference category excluded in the econometric model specification

The *full factorial design* resulted in 96 possible combinations. To avoid cognitive burden and facilitate administration, a *fractional factorial design* was used with 16 forced-choice pair-wise choice sets, ensuring orthogonality (all attributes are orthogonal except for the three-level attribute age that exhibits correlations with the rest of the attribute but all smaller than 0.04), level balance and minimum overlap. For the experimental design Sawtooth Software was used.

### Sample and data collection

The data collection for this study focused on expert stakeholders in the German healthcare sector. An online questionnaire was administered that entailed a detailed description of the survey purpose and guidance on how to interpret and handle the questionnaire. This was followed by 16 choice sets and socio-demographic questions about gender, age, profession, working institution and experience in the healthcare sector (Tables 2, 3).

**Table 2:** Example of a choice set in the DCE questionnaire

Criteria	PROGRAM A	PROGRAM B
Severity of disease	Not severe (LE > 2 years)	Severe (LE < 2 years)
Number of potential beneficiaries	Many (> 100,000)	Few (< 100,000)
Age of target group^*^	Young (0 to 14 years)	Middle-age (15–59 years)
Individual health benefits	Large (**> **5 healthy years)	Small (**< **5 healthy years)
Willingness to subsidise others	Less than 70% of total health expenditure	More than 70% of total health expenditure
Cost-effectiveness	Not cost-effective (Cost / DALY > GDP/cap)	Cost effective (Cost/DALY < GDP/cap)
If you have to fund one of the two programs above which one would you choose?		

^*^With the exception of “Age of target group” all attributes in the experiment comprise two levels. The levels for all two-level attributes are given in the example choice set above. Attribute “Age of target group” comprised three levels, namely, “Young (0–14 years)”; “Middle-age (15–59 years)” and “Old-age (60 years and above)". For presentational purposes and given that our experiment had pair-wise choices, only two of these levels are possible to be represented in the example choice set above

**Table 3:** General demographic descriptive statistics

	# of obs.	Percentage
*Gender*		
Male	185	0.7
Female	78	0.3
*Age*^*a*^		
< 35 years	23	8.85
35–45 years	30	11.54
45–55 years	103	39.62
55–65 years	88	33.85
> 65 years	16	6.15
*Experience*^*b*^		
< 5 years	19	7.28
5–10 years	24	9.20
10–15 years	29	11.11
15–20 years	32	12.26
20–30 years	99	37.93
> 30 years	58	22.22
*Profession*		
Policy maker	83	0.32
Healthcare professionals	100	0.38
Researcher	80	0.30

^a^Calculated with 260 persons since information of 3 respondents is missing

^b^Calculated with 261 persons since information of 2 respondents is missing

Out of 2153 individuals contacted, 263 complete and valid questionnaires were returned giving a response rate of about 12%. The sample targeted individuals involved in the macro-, meso- and micro-levels of healthcare decision-making in Germany and included healthcare academics, members of various legislative and political decision-making bodies accountable for strategy, implementation, funding and supervision, executives of national research and planning institutions, as well as leaders and senior staff members of individual healthcare providers.

### Statistical analysis

Data from respondents who failed to answer all choice sets were dropped. The remaining observations were analysed through a mixed logit regression model (Hole, 2007). This modeling approach allows for multiple observations being obtained from individuals that do not exhibit the restrictive independence from irrelevant alternatives and account for correlations in unobserved heterogeneity of preferences (Hensher & Greene, 2003; Kjær & Gyrd-Hansen, 2008; Revelt & Train, 1998). All coefficients are specified as normally distributed parameters with zero-correlations between random parameters. The DCE model captured the main effects of each domain level. Interaction terms between attributes and individual characteristics (i.e., Sex dummy taking value of 1 if male; Age dummy taking value of 1 if age > 45, Work experience dummy taking value of 1 for > 10 year of experience; and two Profession dummies with reference category Researcher/Academia) were excluded from final model since earlier likelihood-ratio tests on conditional logits found them to be not statistically significant (results remained similar for different individual characteristics threshold values). Similarly, restricting analysis to the policy-makers sub-sample (as the group who is more likely to be involved and influence decision-making) produces very similar results to the full sample analysis and as such sub-group results are omitted (results given in Appendix I).

### Equity/ efficiency profiles and ratios

As the magnitude of estimated parameters cannot be directly interpreted, results are discussed in terms of percentage changes in predicted probabilities for each attribute (Lancsar et al., 2007; Mentzakis et al., 2014; Ryan et al., 2008) as well as for the equity/efficiency groups in aggregate (i.e. summing up all efficiency (or equity) attributes for a fully efficient (or equitable) alternative). Criteria with a higher probability of being chosen will be more likely to influence the selection of the interventions.

Moreover, the difference between the predicted probabilities for the equity-only and efficiency-only interventions were calculated by subtraction as well as the percentage change with respect to a baseline, defined as a hypothetical intervention for which all attributes are set at their sample mean values. The results provide an estimate of the size of contribution of the efficiency and equity components and denote the implicit willingness to trade-off these components with each other. Table 5 presents the (changes in) predicted probabilities. Furthermore, changes and percentage changes in predicted probabilities for the aggregated criteria along with the calculated equity-efficiency trade-off (i.e. calculated as the ratios of percentage changes in predicted probabilities of the aggregate Efficiency over the corresponding aggregate Equity value) were separately measured for each age group: interventions targeting young, middle-age and old groups. For all predicted probabilities 95% confidence intervals are calculated through the Delta method.

### Composite league table

To further operationalize estimation results and place them more aptly in a policy relevant context, a composite league table (CLT) is used for illustration. Health interventions are classified and ranked within the context of country-specific clinical conditions. Each intervention is mapped along the attributes of our experiment (an example is given in the Table notes of Appendix II). The information on the mapping of interventions is based on information used in the epidemiology disease models developed and employed in the CHOICE (*CHO*osing *I*nterventions that are *C*ost-*E*ffective) program of the World Health Organization (WHO). The ‘severity of disease’ and ‘individual benefit’ criteria were decided based on primary/secondary preventive and inpatient/outpatient treatment. Willingness to subsidise others is considered to be equally high across interventions due to universal coverage.

According to the disease burden of high-income countries (Mathers et al., 2008; WHO, 2003), 24 types of interventions were considered (cf. Appendix II), i.e. health interventions across the major disease areas, including control of non-communicable, chronic disease threat and risk factors that are of interest in Germany. The main data sources used to choose the clinical conditions were developed by the WHO and partner communities (Alwan, 2011; Murray & Lopez, 2013; Murray & Lopez, 1998; Whiteford et al., 2013), as well as by national guidelines and statistics issued by the German Ministry of Health and associated organisations (Robert Koch Institut, 2006; Federal Ministry of Health, 2007; Berufsverband Deutscher Psychologinnen & Psychologen, 2012; Lademann & Kolip, 2005; Robert Koch Institut, 2014; NVL, 2015).

Given the mapping of interventions to the six attributes that enter the model and the attribute coefficients obtained from the estimation, the probability of selection of each intervention is calculated, often termed “composite index” score (CIs) in the literature, which measures its priority level as determined by its characteristics (Baltussen & Niessen, 2006; Baltussen et al., 2007). Subsequently, all interventions are rank ordered according to their CIs which produces the final CLT ordering. The aim of the CLT is to identify those interventions that should be prioritized for public reimbursement (high-income countries) or health initiative (low-income countries) (Defechereux et al., 2012).

## Results

Table 4 presents the mixed logit estimates for equity and efficiency attributes among German decision-makers. Magnitude is not directly interpretable and therefore we discuss sign and significance of the coefficients in the first instance; a positive sign suggesting utility increasing characteristics and conversely for a negative sign. With the exception of ‘middle age group’ and ‘willingness to subsidise’, all coefficients were statistically significant at 1% (*p* < 0.01).

**Table 4:** Mixed logit estimation results

	Mean	SD
*Equity attributes*		
Severity of disease		
Severe	1.174***	1.185***
	(0.0918)	(0.0800)
Age of target group:		
Middle	− 0.0170	− 0.158
	(0.0661)	(0.109)
High	− 0.829***	− 0.569***
	(0.0812)	(0.125)
Willingness to subsidise others		
More than 70%	− 0.0377	0.227***
	(0.0495)	(0.0840)
*Efficiency attributes*		
Number of potential beneficiaries		
Many	0.980***	0.784***
	(0.0705)	(0.0785)
Individual Health benefits		
Large	0.954***	0.794***
	(0.0697)	(0.0796)
Cost-Effectiveness		
Cost-effective	0.901***	0.889***
	(0.0767)	(0.0869)
Number of Individuals	263	
Number of observations	8416	
Log likelihood	− 2035.2	
BIC	4196.98	

1. Estimates are based on a mixed logit model with normal distribution specified for each attribute, 2. Dependent variable takes the value of 1 if individual chooses that particular alternative, 3. Every individual contributes a total of 32 observations (16 choice sets with 2 alternatives), 4. *indicates that the coefficient is significant at 10% level of significance (*p* < 0.1), ** indicates that the coefficient is significant at 5% level of significance (*p* < 0.05), 6. ***indicates that the variables are significant at 1% level of significance (*p* < 0.01)

Respondents appear to prefer interventions addressing the young (baseline) over those targeting high age groups. Moreover, interventions requiring public support are not favoured. On the other hand, there seems to be a strong preference towards interventions that target the severely-ill as well as towards interventions with substantial health effects for those treated. Not surprisingly, interventions that are beneficial for a larger proportion of the population and those which prove to be cost-effective are favoured.

Moving on to Table 5, with regards to equity criteria, ‘severity of disease’ increases the probability of selection for an intervention by 7.23% (95% CI 6.23–8.22) as compared to the baseline. All other equity attributes reduce the probability of selection as compared to the baseline. However, the effects for ‘middle aged’ and ‘willingness to subsidise others’ are statistically insignificant. Looking at efficiency criteria, all three criteria exhibit large, significant and positive effects. The probability of selecting interventions that entail substantial health benefits increases by 6.11% (95% CI 5.17–7.05) compared to the baseline, while the corresponding probability for interventions that provide benefits to a larger share of the population is 6.25% (95% CI 5.28–7.21), and 5.83% (95% CI 5.00–6.66) for interventions that are cost-effective. With regards to aggregate criteria along with the calculated equity/efficiency trade-off, interventions appear to be strongly preferred when improving efficiency, independent of the age group that is targeted. This, however, is especially true for interventions targeting young and high age groups. Except for aggregated equity criteria for high age groups, all coefficients are significant.

**Table 5:** Predicted Probabilities from mixed logit, % changes in predicted probabilities, and efficiency/equity trade-off

	Predicted probability ^b^	% Δ compared to baseline ^c^	Interventions targeting young age groups		Interventions targeting middle age groups		Interventions targeting high age groups	
			% Δ for aggregate Efficiency/ Equity	Efficiency/ Equity ratio (trade-off)	% Δ for aggregate Efficiency/ equity	Efficiency/ Equity ratio (trade-off)	% Δ for aggregate Efficiency/ equity	Efficiency/ Equity ratio (trade-off)
Base Alternative ^a^	0.85 (0.82 – 0.87)							
*Equity attributes*								
Severity of disease	0.91 (0.89–93)	7.23 (6.23–8.22)	5.52 (4.81–6.23)		5.47 (4.59–6.34)		− 0.03 (− 2.11–2.05)	
Middle age	0.85 (0.82–0.88)	− 0.17 (− 1.48 –1.14)						
High age	0.76 (0.72–0.80)	− 10.4 (− 13.4 –7.52)						
Willingness to subsidise others	0.85 (0.82–0.87)	− 0.29 (− 1.04 –0.47)						
				1.82 (1.57–2.07)		1.84 (1.54–2.15)		− 340.6 (− 25,881–25,199)
*Efficiency attributes*								
Number of potential beneficiaries	0.90 (0.88–0.92)	6.25 (5.28–7.21)	10.07 (8.60–11.5)		10.09 (8.63–11.54)		9.45 (8.39–10.5)	
Individual health benefits	0.90 (0.87–0.92)	6.11 (5.17–7.05)						
Cost-effectiveness	0.90 (0.88–0.92)	5.83 (5.00–6.66)						

^a^Within the base alternative all attributes are set at their mean

^b^Each alternative is identical to the base, except of the attribute of interest which is set at 1

^c^Predicted probabilities of “Base Alternative”, “Middle age” and “Willingness to subsidise others” appear equal due to rounding to two decimals for the Table. As the three underlying numbers are different, percentage differences (i.e. % Δ) between them are non-zero. However, as expected such differences are highly statistically insignificant

### CLT

Based on the estimated coefficients, an overall ranking is presented in the Appendix II. Several interventions have similar characteristics with respect to our efficiency and equity criteria, resulting in similar scores and, hence, rankings. According to the results of the CLT, interventions aimed at mental disorders and CVDs are among those ranked the highest.

Overall, interventions targeting psychological and behavioural disorders as well as cardiovascular diseases exhibit the highest-ranking scores, closely followed by neoplasms and diabetes (endocrine, metabolic diseases). Intervention “Education, promote individual, family, community connectedness” targeting the condition “Suicide and intentional self-harm” is the highest ranked intervention for the German stakeholders.

## Discussion

This study draws attention on the use of discrete choice experiments to devise rational frameworks for priority setting, taking explicitly into account the concerns for different societal objectives. In this regard, the results of the experiment on a sample of relevant stakeholders in the German health system allow one to discuss some interesting findings.

German decision makers consider severity of disease, individual health benefits, cost-effectiveness, as well as number of potential beneficiaries as important criteria for priority setting. The absolute values of the regressions reflect their relative importance in priority setting. Based on their respective weights, severity of disease, number of potential beneficiaries, individual health benefits and cost-effectiveness appear as the most important criteria for German decision makers within the sample population, displaying great preference towards efficiency.

Besides the general pro cost-effectiveness attitude, respondents associate a higher utility to interventions targeting younger age groups. This is in line with empirical studies in Germany, such as Raspe and Stumpf (2013), which find priority setting in favour of treatments for children. Willingness to subsidise others appears insignificant, which again confirms a priori expectations in high-income countries such as Germany that are characterised by universal coverage (cf. Norway and Austria). Overall, all efficiency attributes are favoured over equity criteria, except for severity of disease (Diederich et al., 2012). Although equity concerns seem to be comparably less important in healthcare resources allocation decisions in Germany, the two objectives (i.e. efficiency and equity) are in conflict with each other and are equally needed in a deliberative process (Culyer, 2006, 2015). The estimated ratios between equity and efficiency support a general preference for efficiency over equity criteria for all age groups, with much stronger results for interventions targeting younger and higher age groups.

These findings show a large overlap with the prioritisation discussion in Germany and are aligned with what was proposed by influential bodies in the German health community. For instance, in a second plea in favour of a priority setting debate in 2007 (first in 2000) the ZEKO addresses the importance of defining the best relative weight for the much-needed prioritisation criteria. Almost all revealed criteria, namely proven benefit/fitness of purpose, cost–effectiveness and medical need (urgency and severity), support our findings. Nevertheless, the general preference assigned to efficiency does not involve a lack of concern for equity. Indeed, basic equity in terms of financial protection is guaranteed through the basic solidarity principle grounded within the SHI. This principle entitles every individual to the same services irrespective of their insurance status or the contributions paid (Deutsche Sozialversicherung, n.d.).

The CLT results can be considered indicative when prioritizing among interventions. Largely, the resulting rankings reflect the National Health Goals (Bundesministerium für Gesundheit, Gesundheitsziele.de, 2022) concerning Type 2 diabetes, breast cancer, depressive disorder, healthy ageing, reduction of alcohol and tobacco consumption and enhancing health competence (Federal Ministry of Health, 2007). These main goals are a complementary governance tool in healthcare and seek to improve the health of individuals or specific groups to tackle the conditions of highest urgency. Yet, the high prioritization of mental disorders is not in line with Schröter and Diederich (2013) who reported that the German population considers mental health of lower importance for prioritisation of medical resources compared with physical health. Nevertheless, such discrepancies in preferences for mental health could largely depend on the specific context.

Together with the National Health Goals, the Information System of the Federal Health Monitoring and the Federal Joint Committee identified the disease burden as one of the most relevant determinants in setting healthcare priorities. This approach is in line with the WHO “2013–2020 Global Action Plan for Prevention and Control of Non-communicable Diseases” that underlines the need to urgently address prevention problems, and to allocate more resources to the early treatment of chronic NCDs and mental illnesses (WHO, 2013).

Similarly to the German results, preferences for efficiency and equity criteria elicited across countries have displayed individual benefits, severity of disease and cost-effectiveness as the most significant priorities in high income countries, HICs (Baji et al., 2016; Defechereux et al., 2012; Mentzakis et al., 2014). In low-income countries (LICs) like Ghana or Nepal (Baltussen et al., 2006, 2007) instead, number of beneficiaries, individual benefits, cost-effectiveness, severity of disease, and middle-aged people are found to be the preferred criteria—showing more balanced equity/efficiency preferences. Results of a Chinese study (Paolucci et al., 2015) disclose a much closer profile resembling that of the mentioned high income countries where universal health coverage is in place.

Comparing the CLTs obtained for Germany with those for Austria and Norway (as instances of comparable HICs) findings are largely comparable (Defechereux et al., 2012; Mentzakis et al., 2011). In those studies, countries share a similar disease burden, comprising mainly mental disorders and NCD, including diabetes, cancer, and cardiovascular diseases. This holds true for the CLT that assigns relatively high rankings for respective disease areas. Compared to other HICs, German decision makers seem to rank higher those interventions affecting young or middle-aged people.

One of the limitations of DCEs is their hypothetical nature. Due to the explorative nature of the study the findings cannot be directly implemented into national policy making but could act as a first step and guide. In fact, results can contribute towards the German debate on setting priorities in healthcare. Further, we note that while our sample size is not small and allows for robust estimation, the low response rate suggests caution in inference and limited generalizability, while future research should explore the congruence of preference between stakeholders and general public. Nonetheless, the methodology is generalizable and can be transported to other countries and settings when the required conditions for successful multi-criteria decision analysis (MCDA) in health are met. Apart from age, non-linear effects were not incorporated in the analysis. Our design identifies individuals’ direction of preferences rather than the exact shape of their function, while future research could focus on non-linearities. The survey and attribute levels were taken from a larger DCE project targeting many countries and as such reference levels in the dichotomization of the attributes were taken to meet international standards and ensure cross-country comparability. Yet, such dichotomization and use of attributes with different scales could introduce vagueness and affect their perception by respondents and conceal potential difference in the relative importance of attributes. Future studies could increase the number of levels for relevant attributes and obtain preferences over a range of discrete attribute values.

## Conclusion

Establishing criteria for equitable and efficient resource allocation in healthcare is a political task with a number of dimensions including medical, economic, ethical and legal ones. The complexity of the issue makes it impossible to achieve a complete consensus between all those involved. Nevertheless, principles ought to be formulated in which existing structures and processes must be measured, not at least in the sense of a future-oriented perspective.

In conclusion, this explorative study details how multiple criteria can guide a transparent and systematic priority-setting process by allowing for the simultaneous assessment of multiple policy objectives of decision-makers. With German decision makers stating a preference for efficiency, such an approach can help to support the priority setting processes and may contribute to a more informed and participated debate on priority setting between different health interventions in Germany.

## Appendix I Policy Makers estimation results

### Mixed logit estimation results for the Policy Makers subgroup

**Table:**

	Mean	SD
*Equity attributes*		
Severity of disease	0.965***	1.410***
	(0.183)	(0.175)
Age of target group		
Middle	0.202*	− 0.146
	(0.119)	(0.241)
High	− 0.744***	1.022***
	(0.161)	(0.222)
Willingness to subsidise others	0.115	0.573***
	(0.0994)	(0.167)
*Efficiency attributes*		
Number of potential beneficiaries	1.098***	0.812***
	(0.128)	(0.138)
Individual health benefits	0.907***	0.703***
	(0.118)	(0.152)
Cost-effectiveness	0.743***	0.922***
	(0.120)	(0.163)
Number of individuals	83	
Number of observations	2656	
Log likelihood	− 652.66812	
BIC	1415.72	

1. Estimates are based on a mixed logit model with normal distribution specified for each attribute, 2. Dependent variable takes the value of 1 if individual chooses that particular alternative, 3. Every individual contributes a total of 32 observations (16 choice sets with 2 alternatives), 4. *indicates that the coefficient is significant at 10% level of significance (*p* < 0.1), **indicates that the coefficient is significant at 5% level of significance (*p* < 0.05), 6. ***indicates that the variables are significant at 1% level of significance (*p* < 0.01)

## Appendix II Composite league table

**Table:**

Intent	Clinical condition	Intervention	CIs	Rank
Psy	Suicide and intentional self-harm	Education, promote individual, family, community connectedness	0.9810	1
CVD	Congestive heart failure	Surgery (coronary artery bypass graft)	0.9582	2
CVD	AMI (Acute myocardial infarct)	Medication (aspirin, atenolol, streptokinase, rt-PA)	0.9575	3
CVD	AMI (Acute myocardial infarct)	Surgery (primary angioplasty, primary stenting, PTCA)	0.9575	3
CVD	Angina pectoris (stable angina)	Angioplasty, stenting	0.9575	3
CVD	Angina pectoris (stable angina)	Surgery (coronary artery bypass graft)	0.9575	3
CVD	Atherosclerosis	Medication (aspirin, atenolol, ACE inhibitors, statins)	0.9575	3
CVD	Atherosclerosis	Surgery (PTCA)	0.9575	3
Neo	MN of the female breast	Surgery (lumpectomy, mastectomy) with adjuvant treatment	0.9575	3
Neo	MN of colon rectum, anus	Surgery with/without adjuvant treatment	0.9575	3
Neo	MN of prostate	Surgery with/without adjuvant treatment	0.9575	3
Endo	Diabetes mellitus type 2	Foot care (patient and provider education, foot examination, foot hygiene, appropriate footwear)	0.9575	3
Endo	Diabetes mellitus type 2	Glucose and blood pressure control (insulin injection, oral glucose- lowering agents)	0.9575	3
Psy	Unipolar depressive disorder	Older antidepressant drug medication (TCA)	0.9575	3
Psy	Unipolar depressive disorder	Newer antidepressant drug medication	0.9575	3
Psy	Unipolar depressive disorder	Psychosocial treatment	0.9575	3
Musc	Lumbar disc herniation	Surgery—microdisectomy	0.9575	3
Neo	MN of prostate	Monitor cancer (watchful waiting, active surveillance)	0.8967	18
CVD	Cerebrovascular disease (acute)	Medication (aspirin, heparin, rt-PA)	0.8942	19
Neo	MN of the larynx and trachea, bronchus, lung	Surgery with/without adjuvant treatment	0.8942	19
Resp	COPD, stage 3–4	Home oxygen therapy	0.8942	19
CVD	Congestive heart failure	Medication (ACE inhibitors, beta-blockers)	0.8763	22
Psy	AD & dementias (stage 1)	Comprehensive in-home care	0.8763	22
CVD	Angina pectoris (stable angina)	Medication (atenolol, ACE inhibitors, beta-blockers)	0.8744	24
CVD	High blood cholesterol	Medication (statins)	0.8744	24
CVD	Hypertension	Medication (ACE inhibitors, beta-blockers)	0.8744	24
Neoplasia	MN of the female breast	Screening (50–70 years) (biennial mammography)	0.8744	24
Neoplasia	MN of colon rectum, anus	Screening (FOBT, colonoscopy, sigmoidoscopy)	0.8744	24
Neoplasia	MN of prostate	Screening (DRE, PSA test)	0.8744	24
Endocrinology	Diabetes mellitus type 2	Education (patient self-management)	0.8744	24
Resp	COPD, Stage 1–2	Medication (inhaled ipratropium bromide, rapid- acting bronchodilators, inhaled corticosteroid)	0.8744	24
Resp	Asthma bronchial control	Medication (inhaled ipratropium bromide, rapid- acting bronchodilators, inhaled corticosteroid, beta-2 agonists)	0.8744	24
Muscular and Skeleton	Lumbar disc herniation	Non-surgical treatment (physiotherapy, osteopathy, steroids)	0.8744	24
Lifestyle	Unhealthy diet	Reduce salt intake	0.8744	24
Lifestyle	Unhealthy diet	Promote public awareness about diet	0.8744	24
Lifestyle	Unhealthy diet	Promote healthy eating in schools	0.8683	36
Lifestyle	Physically inactivity	Promote physical activity in schools	0.8683	36
Lifestyle	Unhealthy diet	Provide health education in worksites	0.8663	38
CVD	Congestive heart failure	Surgery (heart transplant)	0.7775	39
Resp	COPD, Stage 3–4	Surgery (lung volume reduction, lung transplant)	0.7745	40
CVD	Cerebrovascular disease (prevention and recurrence)	Medication (aspirin, dipyridamole, carotid endarterectomy)	0.7651	41
Psy	AD & dementias (stage 2)	Nursing home/hospital care	0.7421	42
Lifestyle	Physically inactivity	Promote physical activity in mass media	0.7285	43
Lifestyle	Tobacco use	Raise tax on tobacco	0.7285	43
Lifestyle	Tobacco use	Enforce clean indoor air law	0.7233	45
Lifestyle	Harmful alcohol use	Raise tax on alcohol	0.7233	45
Lifestyle	Physically inactivity	Offer counselling in primary care	0.5215	47
Lifestyle	Harmful alcohol use	Enforce drink-driving laws (breath-testing)	0.5150	48
Lifestyle	Tobacco use	Enforce bans on tobacco advertising/public smoking places	0.5018	49
Lifestyle	Harmful alcohol use	Enforce bans on alcohol advertising	0.5018	49

*Psy* psychological and behavioural disorders*, CVD* cardiovascular diseases, *Resp* respiratory diseases, *Endo* endocrine, nutrition and metabolic disorders, *Musc* diseases of the muscular and skeletal system, *Neo* (malignant) neoplasms, *Lifest* risk-related factors/Lifestyle

Construction of the CLT begins by taking each intervention under consideration and assigning it values for each attribute. For example, an intervention to “Promote healthy eating in school” would take the value of 0 for Severity (i.e. low severity), value of 1 for Individual Benefit (i.e. large individual benefits), value of 0 for Middle-age and value of 0 for Old-age (ie as it target young people), value of 0 for cost-effectiveness (i.e. low cost-effectiveness), value of 1 for Number of potential beneficiaries (ie high number of beneficiaries) and value of 1 for willingness-to-subsidize others (i.e. more than 70%). Following such mapping and given the attribute coefficients (ie part worth utilities) obtained from the model estimation, predicted probabilities for each intervention can be computed indicating the probability with which an intervention would be chosen to be funded. Ranking interventions by their probabilities produces the CLT ranking

## Footnotes

1 Commissions working on prioritisation in other countries are: Norway (Lønning Committee I und II, 1987/1997), Orgeon, USA (Oregon Health Service Commission, 1989), Netherlands (Dunning Committee, 1992/1995), Sweden (Commission of Parliament and Experts, 1993/1995), New Zealand (Core Services Committee/National Health Committee, 1993), Israel (Medical Technical Forum and National Advisory Committee, 1995), Denmark (Danish Counsil of Ethics, 1997) and England, Wales (National Institute for Health and Clinical Excellence [NICE], 1999).
